# *De novo* domestication towards new crops

**DOI:** 10.1093/nsr/nwab033

**Published:** 2021-02-26

**Authors:** Xianrong Xie, Yao-Guang Liu

**Affiliations:** State Key Laboratory for Conservation and Utilization of Subtropical Agro-Bioresources, South China Agricultural University, China; Guangdong Laboratory for Lingnan Modern Agriculture, China; College of Life Sciences, South China Agricultural University, China; State Key Laboratory for Conservation and Utilization of Subtropical Agro-Bioresources, South China Agricultural University, China; Guangdong Laboratory for Lingnan Modern Agriculture, China; College of Life Sciences, South China Agricultural University, China

Long-term natural and human selections have domesticated crops from their wild progenitors for millennia, leading to the fixation of a large number of agronomically beneficial alleles and traits in cultivated crops. However, this process has been extremely time-consuming, accompanied with negative consequences, such as genetic erosion, accumulation of deleterious mutations and decreased environmental adaptation. Global population increase and climate change are increasingly threatening food security. One potential solution to this problem is the use of genome editing for rapid domestication of novel crops [1]. Pioneers in this field have managed to *de novo* domesticate wild tomatoes and groundcherry [2,3]. In a recent issue of *Cell*, Li's group from the Chinese Academy of Sciences and collaborators provided an exciting illustration of *de novo* domestication of a wild allotetraploid rice [4].

Compared to Asian cultivated rice (*Oryza sativa* with AA genome), the wild allotetraploid relatives have significant advantages in biomass and resistance, but they cannot be used as crops due to their undesirable characteristics, including sprawling growth habit, high photoperiod sensitivity, tall architecture, sparse panicle, long awn, easy seed shattering and low grain yield and quality. To develop wild tetraploid rice as a modern crop, the group established a four-step strategy to modify these undesirable traits: (i) selecting a wild allotetraploid material suitable for domestication; (ii) developing an efficient transformation system; (iii) assembling and annotating a high-quality reference genome; and (iv) editing several domestication-related and agronomically important genes to improve various traits (Fig. 1). By investigating callus induction and regeneration abilities as well as growth phenotypes for 28 lines of wild allotetraploid (CCDD genomes) species, they selected an *O. alta* line as the starting material, and established an efficient transformation method. Then, a high-quality reference genome of this line was generated. The genome could be divided into two subgenomes (C_t_ and D_t_) that are significantly distinct from the AA genomes of this genus.

**Figure 1.**
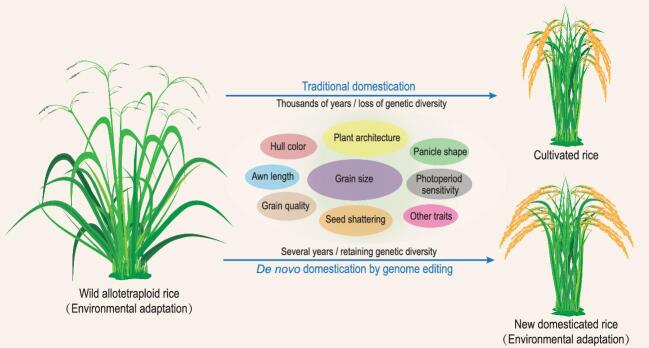
*De novo* domestication of wild plants towards new crops. By genome editing of key genes for domestication and agronomic traits, wild allotetraploid rice can be rapidly domesticated into new-type rice cultivars that have strong environmental adaptability and high grain yield.

Previous literature regarding domestication and agronomic genes in rice inspired the authors to target the corresponding orthologues in *O. alta* using CRISPR-Cas. They firstly knocked out the *qSH1* and *An-1* orthologues conferring seed shattering and awn length respectively, resulting in a significantly lower rate of seed shattering and a shorter awn length in the mutants. They further edited several *O. alta* orthologues of rice genes for plant height (*SD1*), ideal plant architecture (*IPA1*), grain length and size (*GS3*), and heading date (*Ghd7*, *DTH7*) to obtain mutant lines with improved traits. Finally, the group created *O. alta* mutant lines with multiple genes edited for these combined traits.

Previous attempts to improve agronomic traits by genome editing were focused on modulating related genes in cultivars rather than in wild plants, which can make incremental improvements on the genetic backgrounds of crops, but cannot exploit the potential of using wild species for breeding. Adding to the previous studies [2,3], this work further illustrates the potential of *de novo* domestication of wild plants in generating brand new crops. Unlike traditional domestication processes, *de novo* domestication maintains genetic diversity and elite traits (such as high environmental adaptability and high biomass) of the wild plants while improving the yield productivity. Moreover, because many wild plants (including the wild *Oryza* species) are perennial, commercial cultivation of new crops domesticated from such plants would likely revolutionize the cropping system, dramatically increasing the efficiency of agriculture production.

Many agronomic traits are controlled by major genes, quantitative trait loci (genes) and epigenetic modifications of genomic DNA, chromatin and RNA. However, for most wild plants (especially polyploidy plants) we have little or no knowledge on the relevant genetic/epigenetic pathways, which may largely differ from those in the well-studied model plants. Thus, to achieve the long-term goal of *de novo* domestication of wild plants, many challenges remain, especially in decoding the genetic/epigenetic basis of beneficial agronomic traits in crops and wild plants, and integrating functional genomic discoveries with genome editing designs. On the other hand, to optimize most agronomic traits, besides gene-knockout, fine-scale subtle changes of gene expression, via editing of their coding and regulatory sequences, are often required [5]. Therefore, different genome editing tools and strategies are needed for modifying target genes in various crops and wild plants. Although more genetic trait improvements for the *O. alta* editing lines are required for generating a commercially usable crop, this work sheds light on the potential to rapidly create novel crops from wild plants using advanced biotechnologies.

## FUNDING

This work was supported by grants from the National Natural Science Foundation of China (31991222) and the Major Program of Guangdong Basic and Applied Basic Research (2019B030302006).

***Conflict of interest statement*.** None declared.
